# Characteristics of individuals with tuberculosis in an urban, poor population in Osaka City, Japan — a case-control study

**DOI:** 10.5365/wpsar.2018.9.1.005

**Published:** 2020-03-31

**Authors:** Akira Shimouchi, Yuko Tsuda, Jun Komukai, Kenji Matsumoto, Hideki Yoshida, Akihiro Ohkado

**Affiliations:** a Nishinari District Public Health Office, Osaka City, Japan.; b Osaka City Public Health Office, Japan.; c The Research Institute of Tuberculosis, Japan Anti-tuberculosis Association, Tokyo, Japan.

## Abstract

**Objective:**

To identify individual characteristics related to the development of pulmonary tuberculosis (PTB) among residents in the Airin area (Airin), Osaka City, Japan.

**Methods:**

We conducted a retrospective case-control study of individual characteristics potentially related to the development of PTB by comparing PTB patients and residents without tuberculosis (TB) in Airin. The following binominal data of characteristics were compared: age (< 65 or  > 65); body mass index (BMI) (< 18.5 or  > 18.5); diabetes mellitus (diagnosed or not diagnosed); smoking (currently smoking any amount or not smoking); and alcohol use (currently drinking any amount or not drinking).

**Results:**

We compared the individual characteristics of 192 PTB patients notified from January 2015 to December 2018 and 190 residents of supportive houses who attended a health education programme from April 2016 to March 2018.

Univariable analysis showed that the following characteristics were significantly related with PTB: BMI < 18.5 (odds ratio [OR]: 6.54, 95% confidence interval [CI]: 3.58–11.97, *P* < 0.001) and current alcohol use (OR: 1.88; 95% CI: 1.24–2.85, *P* = 0.003). Multivariable analysis showed similar results: BMI < 18.5 (adjusted odds ratio [aOR]: 6.90, 95% CI: 3.72–12.79, *P* < 0.001) and current alcohol use (aOR: 2.15, 95% CI: 1.36–3.42, *P* = 0.001).

**Discussion:**

Undernutrition and alcohol use are individual characteristics associated with PTB among residents in Airin, Osaka City. To strengthen the TB control programme further, it is suggested to develop new programmes for primary prevention.

Osaka City had the highest tuberculosis (TB) notification rate (32.4 per 100 000) of any city in Japan (national average: 13.3 per 100 000) in 2017. (*1*) The rate is particularly high in the densely populated Airin area, where 21 500 residents live in 0.62 km2. In 2017, 88 TB patients were notified (409 notifications per 100 000 people). (*2*) According to a 2015 city government survey, 43% of the residents of Airin are enrolled in a public assistance programme, 52% are pensioners and daily-paid labourers without public assistance and some without health insurance, and 5% are homeless individuals living in shelters, on the streets or in parks. (*2*)

Public assistance is provided for people who are unable to maintain a minimum standard of living. The recent increase in the number of persons enrolled in the public assistance programme in Airin is due in part to the ageing of daily-paid labourers. (*3*) To apply for public assistance, an individual or supporter must meet the following eligibility conditions: (i) there are no savings, insurance, assets or property; (ii) monthly income is below the local minimum wage; and (iii) there are no relatives to provide financial support. People who meet those conditions are entitled to receive a standard amount of cash, which is close to the local minimum wage. Among other services, free medical services are available for all persons on public assistance, as long as a medical board agrees to the appropriateness of each treatment during a monthly review. With regard to the housing condition in Airin, 89% of residents lived in apartments, the majority of which did not have a toilet or bath in each room, and 11% lived in detached houses or semi-detached houses in 2005. (*4*) Since then, the number of apartments has increased, but reports on housing conditions specific to Airin have not been published.

Certain risk factors can impact an individual’s vulnerability to developing TB. Reported risk factors for TB can be divided into two groups: socio-environmental factors (e.g. lack of education, low income, unemployment, overcrowding and poor ventilation in the houses) and individual characteristics (e.g. 65 years and older, tobacco use, alcohol use, diabetes mellitus, undernutrition and weak immune status). (*5*-*10*)

In Airin, the TB control programme implemented by the Osaka City government has so far emphasized active case finding by chest X-ray and patient support. Free medical services and residences are provided for persons who are low income or homeless, in collaboration with the district social welfare office. (*11*) To strengthen the TB programme further, we consider that ascertaining the individual characteristics of the population at the highest risk of developing TB would lead to more effective health education programmes for the prevention and early detection of TB.

This study aims to identify individual characteristics related to the development of pulmonary TB (PTB) among residents in Airin, Osaka City, Japan.

## Methods

We conducted a retrospective case-control study of individual characteristics potentially related to the development of PTB by comparing PTB patients and residents without TB in Airin, Osaka City, Japan.

### Selection of cases

Cases were PTB patients registered in Airin from January 2015 to December 2018. We excluded patients with extrapulmonary TB in this analysis because they are bacteriologically confirmed less frequently than PTB patients. Furthermore, the clinical diagnosis of extra-pulmonary TB, especially TB pleuritis (pleural effusion as a chest X-ray finding) is less certain than that of PTB. All TB patients are diagnosed at medical facilities and reported to the Osaka City Public Health Office. The TB Diagnostic Committee of Osaka City confirms PTB diagnoses by reviewing radiologic (chest X-ray with or without computed tomography [CT] films) bacteriological and clinical test results. After a TB patient is registered, public health nurses (PHNs) in each district provide individualized support until treatment completion. Patients are either bacteriologically confirmed or clinically diagnosed. Bacteriologically confirmed PTB is defined as the presence of *Mycobacterium tuberculosis* in sputum using smear, culture, and identification by immuno-chromatography or nucleic acid amplification methods. Clinically diagnosed PTB is defined as a physician’s decision based on clinical evidence, such as a chest X-ray, CT and interferon-gamma release assays (IGRA). Lastly, we excluded homeless PTB patients because it is too difficult to obtain agreement to collect data on individual characteristics of homeless persons as controls within the framework of current TB control activities.

### Data collection for PTB patients

To collect data on the targeted characteristics of PTB patients, PHNs used structured patient cards developed and updated by the Osaka City Public Health Office. Printed on each card was a list of signs and symptoms such as cough, fever, haemoptysis, general malaise, loss of body weight, difficulty breathing, chest pain and loss of appetite. For each PTB patent, PHNs looked for signs and symptoms. If a patient showed any sign or symptom, PHNs asked when the sign or symptom started and recorded the date accordingly. Smoking and drinking habits were self-reported. Diabetes mellitus was diagnosed by a physician without distinction of type 1 or 2. PHNs measured body weight and height of the patient at the initial interview. PTB patients who did not have a complete data set for targeted characteristics were excluded to increase the reliability of the statistical analysis.

Body mass index (BMI) is a measure of body weight (kg) divided by height (m). Low BMI is known as a proxy indicator of malnutrition, contributing to TB development. However, as body weight loss is also a typical symptom of TB, low BMI could be a result of TB disease. (*12*) Therefore, frequency of low BMI would be overestimated as a risk factor of TB when patients with body weight loss are included. Thus, to assess BMI before TB developed, we divided TB patients into two groups: patients with body weight loss before TB diagnosis and patients without body weight loss.

### Selection of controls

Controls were selected from among non-PTB residents in eight “supportive houses” in Airin. (*13*) The living conditions of supportive houses – one-room apartments without bath or toilet – were similar to those of the apartments housing the PTB patients enrolled in the study – small, single rooms (4.6 m^2^) without toilet or kitchen. An exception is that supportive houses have staff who help residents in aspects of daily life. This may include assistance in attending clinics/hospitals and administration of prescribed drugs, as needed. In addition, they have a common room for residents where PHNs can hold health education sessions.

### Data collection for controls

From April 2016 to March 2018, PHNs compiled data on the same targeted characteristics of case controls as was used for the case patients, taking advantage of health education sessions held every two months in supportive houses. Among the controls, diagnosis of diabetes mellitus was self-reported without a physician’s confirmation. To reduce the possibility of a control having PTB without symptoms, PHNs asked about the history of chest X-ray screening and attendance at medical facilities in the previous year.

### Defining study group characteristics

From the collected data, binomial risk factor data were defined as follows: age (< 65 or  > 65); BMI (< 18.5 or  > 18.5); diabetes mellitus (diagnosed or not); smoking (currently smoking any amount or not smoking); and alcohol use (currently drinking any amount or not drinking). PHNs recorded these data on an electronic spreadsheet.

### Analysis

Univariable analysis was done to make sure that there was no statistical difference of frequency of characteristics between bacteriologically confirmed PTB patients and clinically diagnosed ones.

To ascertain association between targeted characteristics and TB disease, univariable and multivariable logistic regression analyses were used to calculate the odds ratio (OR) and adjusted odds ratio (aOR), respectively, and their corresponding 95% confident interval (CI). We applied backward stepwise selection for multivariable analysis. A *P*-value of less than 5% was considered statistically significant. All statistical analyses were performed using SPSS version 11.0J for Windows (SPSS Inc., Chicago, IL, USA).

### Ethical considerations

The Ethical Review Committee of the Research Institute of Tuberculosis, Japan Anti-tuberculosis Association, Tokyo, Japan, approved the study (authorization number: RIT/IRB 29–12).

## Results

**Figure 1:**
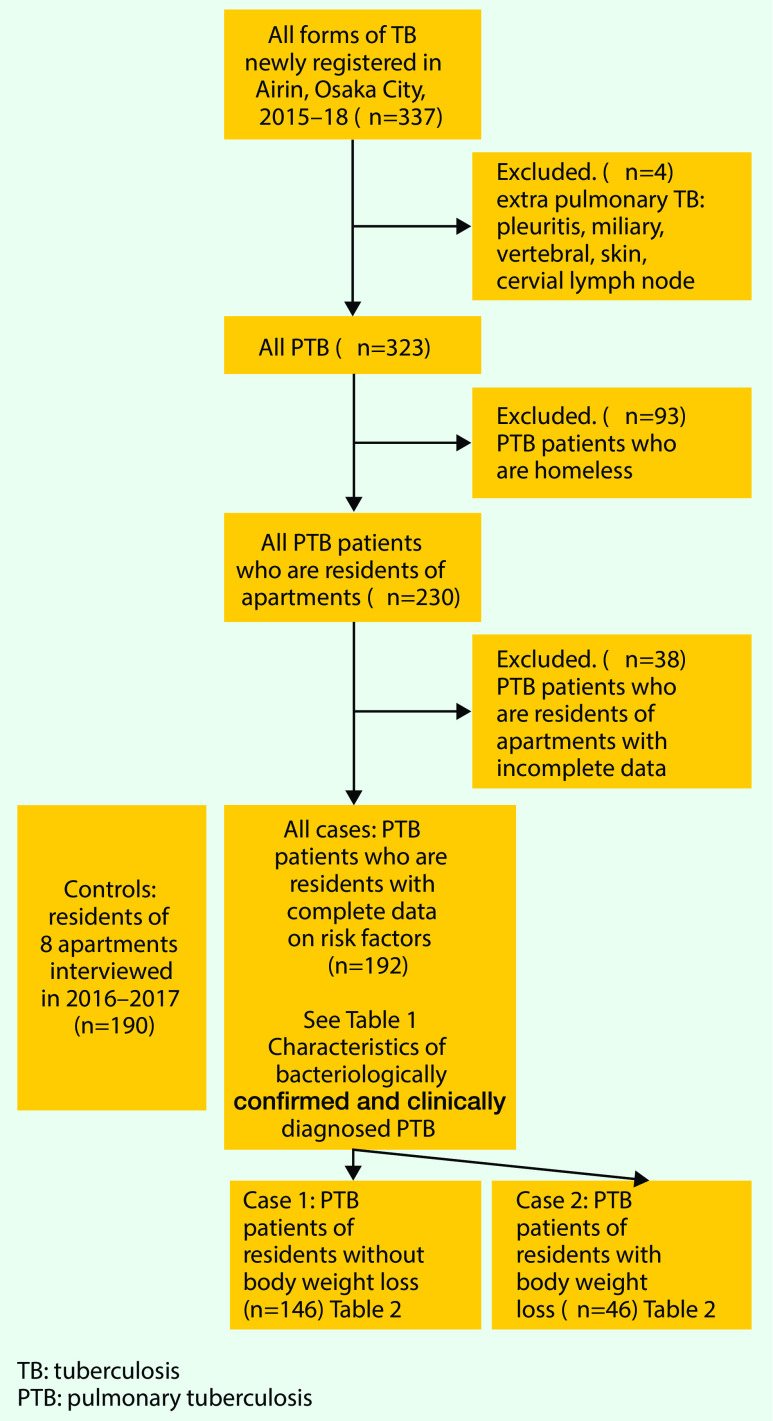
**Study Profile**

Overall, 337 TB patients were registered in Airin from January 2015 to December 2018. There were 14 patients with extrapulmonary TB (TB pleuritis: 7, miliary TB: 2, vertebral TB: 2, skin TB: 1, cervical lymphadenitis: 2). Of the 323 PTB patients, 93 were homeless and therefore excluded from the analysis, and 230 were residents of either one-room apartments or semi-detached houses (both had rental agreements with rents of semi-detached houses slightly higher). Of the 230 PTB patients living in one-room apartments or semi-detached houses, 38 PTB patients with incomplete data sets were further excluded. We distinguish two types of residence: one-room apartment and semi-detached house with a few rooms. All controls were living in one-room apartments, while four of the 192 PTB patients with complete data sets were living in semi-detached houses (**Fig. 1**).

[**insert** Figure 1]

Of the 192 PTB patients, 141 were on public assistance, and 51 were not on public assistance. There was no statistical difference in frequencies of characteristics between these two groups except for sex and age. PTB patients not on public assistance had higher rates of females (18% vs 3%) and persons aged < 65 (55% vs 25%) than PTB patients on public assistance. There was no statistical difference in frequencies of characteristics between bacteriologically confirmed PTB patients and clinically diagnosed PTB patients (**Table 1**).

**Table 1:** Comparison of characteristics of bacteriologically confirmed PTB and clinically diagnosed PTB among residents in Airin, Osaka City, 2015–2018

-	Bacteriologically confirmed TB (*n* = 147)	Clinically diagnosed TB (*n* = 45)	Univariable analysis	*P*-value
-	*n*(%)	*n*(%)	Odds ratio (95%CI)	-

Sex				
Male	136 (93)	43 (96)	0.74 (0.15–3.79)	0.72
Female	11 (7)	2 (4)	1	-

Age				
< 65	50 (34)	14 (35)	1.20 (0.56–2.61)	0.64
> 65	97 (66)	31 (65)	1	-

BMI kg/m^2^				
< 18.5	56 (38)	13 (29)	1.21 (0.56–2.63)	0.63
> 18.5	91 (62)	32 (71)	1	-

^Diabetes mellitus^				
Yes	32 (22)	10 (22)	0.88 (0.37–2.09)	0.77
No	115 (78)	35 (78)	1	-

^Smoking^				
Yes	79 (54)	29 (64)	0.59 (0.27–1.29)	0.19
No	68 (46)	16 (36)	1	-

^Alcohol^				
Yes	68 (46)	20 (44)	1.26 (0.59–2.67)	0.55
No	79 (54)	25 (56)	1	-

PTB: pulmonary tuberculosis

PHNs interviewed 215 residents in eight supportive houses to determine eligibility for inclusion as controls. Two residents receiving ongoing TB treatment were excluded. The interviews indicated that the prevalence of TB was 2/215 (0.93%) or 930 per 100 000 population, which is higher than the TB notification rate in the entire Airin area. Subsequently, 23 residents with incomplete data on characteristics were excluded. The remaining 190 persons with complete data sets for targeted characteristics were chosen as controls. Of them, 170 persons were on public assistance, and 20 persons were not on public assistance. There was no statistical difference in frequencies of characteristics between these two groups. Of the 190 controls, 162 (85%) regularly attended medical clinics to receive care for chronic illnesses such as hypertension, liver disease, heart disease and diabetes mellitus, and 134 (71%) had a chest X-ray examination at a clinic or the TB screening programme within one year. In total, 180 (95%) had attended medical clinics or had a chest X-ray screening within one year. As of July 2019, none of the controls was notified as a TB patient in Airin.

For the 192 PTB patients, univariable analysis showed that the following characteristics were significantly related with PTB: BMI < 18.5 (OR: 6.54; 95% CI: 3.58–11.97, *P* < 0.001) and current alcohol use (OR: 1.88, 95% CI: 1.24–2.85, *P* = 0.003). Multivariable analysis showed similar results: BMI < 18.5 (aOR: 6.90, 95% CI: 3.72–12.79, *P* < 0.001) and current alcohol use (aOR: 2.15, 95% CI: 1.36–3.42, *P* = 0.001). In the same way, for the 146 PTB patients without body weight loss, univariable analysis and multivariable analysis showed that following characteristics were significantly related with PTB: BMI < 18.5 (OR: 4.87, 95% CI: 2.58–9.20, *P* < 0.001) and current alcohol use (OR: 1.94, 95% CI: 1.24–3.03, *P* = 0.004), and BMI < 18.5 (aOR: 5.00, 95% CI: 2.62–9.52, *P* < 0.001) and current alcohol use (aOR: 2.00, 95% CI: 1.26–3.20, *P* = 0.004), respectively. Lastly, for PTB cases with body weight loss, univariable analysis showed that BMI < 18.5 was related with PTB (OR: 15.17, 95% CI: 6.91–33.29, *P* < 0.001), and multivariable analysis showed that BMI < 18.5 (aOR: 19.48, 95% CI: 8.26–45.93, *P* < 0.001) and diabetes mellitus (aOR: 3.45, 95% CI: 1.44–8.26, *P* = 0.005) were significantly related with PTB (**Table 2****)**.

**Table 2:** Characteristics associated with PTB patients in comparison with residents in Airin area, Osaka City, 2015–2018

-	Controls (*n* = 190)	Total cases (*n* = 192)	Univariable analysis		Multivariable analysis		Cases without body weight loss (*n* = 146)	Univariable analysis		Multivariable analysis		Cases with body weight loss (*n* = 46)	Univariable analysis		Multivariable analysis	
-	*n*(%)	*n*(%)	OR(95%CI)	*P*-value	aOR(95%CI)	*P*-value	*n*(%)	OR(95%CI)	*P*-value	aOR(95%CI)	*P*-value	*n*(%)	OR(95%CI)	*P*-value	aOR(95%CI)	*P*-value
Sex																
Male	182(96)	179(93)	0.60 (0.24–1.50)	0.277	-	-	136(93)	0.60 (0.23–1.55)	0.291	-	-	43(93)	0.63 (0.16–2.47)	0.508	-	-
Female	8(4)	13(7)	1	-	-	-	10(7)	1	-	-	-	3(7)	1	-	-	-

Age																
< 65	56(30)	64(33)	1.20 (0.78–1.84)	0.417	-	-	49(34)	1.21 (0.76–1.92)	0.423	-	-	15(33)	1.16 (0.58–2.31)	0.678	-	-
≥ 65	134(71)	128(67)	1	-	-	-	97(66)	1	-	-	-	31(67)	1	-	-	-

BMI, kg/m2																
< 18.5	15(8)	69(36)	6.54 (3.58–11.97)	< 0.001	6.90 (3.72–12.79)	< 0.001	43(29)	4.87 (2.58–9.20)	< 0.001	5.00 (2.62–9.52)	< 0.001	26(57)	15.17 (6.91–33.29)	< 0.001	19.48 (8.26–45.93)	< 0.001
≥ 18.5	175(92)	123(64)	1	-	1	-	103(71)	1	-	1	-	20(43)	1	-	1	-

Diabetes mellitus																
Yes	37(20)	42(22)	1.16 (0.71–1.90)	0.563	1.57 (0.92–2.66)	0.096	27(18)	0.94 (0.54–1.63)	0.820	-	-	15(33)	2.00 (0.98–4.08)	0.057	3.45 (1.44–8.26)	0.005
No	153(81)	150(78)	1	-	1	-	119(82)	1	-	-	-	31(67)	1	-	1	-

Smoking																
Yes	120(63)	108(56)	0.75 (0.50–1.13)	0.169	0.68 (0.43–1.07)	0.096	83(57)	0.77 (0.49–1.19)	0.242	-	-	25(54)	0.69 (0.36–1.33)	0.272	-	-
No	70(37)	84(44)	1	-	1	-	63(43)	1	-	-	-	21(46)	1	-	-	-

Alcohol																
Yes	59(31)	88(46)	1.88 (1.24–2.85)	0.003	2.15 (1.36–3.42)	0.001	68(47)	1.94 (1.24–3.03)	0.004	2.00 (1.26–3.20)	0.004	20(43)	1.71 (0.88–3.30)	0.111	1.97 (0.90–4.35)	0.091
No	131(69)	104(54)	1	-	1	-	78(53)	1	-	1	-	26(57)	1	-	1	-

Controls: Residents in supportive houses, apartments with staff taking care of residents in daily life, interviewed in 2016–18.

BMI: body mass index

OD: Odds ratio, aOD: adjusted Odds ratio

SPSS version 11.0J for Windows (SPSS Inc., USA) was used for univariable analysis and multivariable analysis.

Multivariable analysis: backward stepwise method is used including all factors in the beginning.

## Discussion

Our research revealed two individual characteristics related to PTB among residents of Airin: BMI < 18.5 and current alcohol use. Most of the cases and controls were in marginal financial condition, i.e. at local minimum wage level. Therefore, it seems that when they spend more money for alcohol, they tend to neglect nutrition.

There was no statistical difference in the proportion of any of the characteristics between patients with bacteriologically confirmed PTB and clinically diagnosed PTB (**Table 1**). It would infer that the quality of the clinical diagnosis of PTB in the study is reliable. Therefore, it would be proper to combine both PTB patient groups to increase the number of cases to obtain significant statistical results.

Malnutrition impairs cell-mediated immunity, which increases vulnerability to specific infectious diseases, (*13*, *14*) including TB. BMI, skinfold thickness and cross-sectional arm muscle area have been used as indicators of malnutrition and are considered reliable. (*10*) However, among them, BMI is the most easily measured, most standardized, and most frequently used in large, population-based cohort studies in many countries, including in Japan. (*15*-*18*) In these cohort studies, BMI of < 18.5 was shown to be a good proxy indicator of undernutrition. Low BMI has been related with higher TB incidence rates in the United States of America (*8*) and the Republic of Korea, (*19*) countries that are socioeconomically close to Japan.

When we measure the effect of low BMI (a sign of undernutrition) on the development of TB, neither case-control nor cross-sectional studies are suitable because they cannot distinguish the direction of the cause–effect relationship. One strength of this study is that we separated PTB patients without body weight loss from those with body weight loss as a TB symptom. By doing this, we could exclude TB-related weight loss and examine BMI solely as a baseline condition before the development of TB, even if it is not perfect. As a result, low BMI was related with PTB in both patients without body weight loss as well as patients with body weight loss. Thus, low BMI was confirmed to be an important factor for the development of PTB.

Diabetes mellitus is only in association with PTB patients with body weight loss. However, as body weight loss is a typical sign of diabetes mellitus, selection of PTB patients with body weight loss seemed to make PTB patients with diabetes mellitus over-present. As diabetes mellitus is not statistically related with PTB patients without body weight loss, we do not consider diabetes mellitus as a risk to develop PTB among residents in Airin.

Alcohol is often consumed in confined spaces with others, such as bars, which may lead to increased exposure to TB patients. (*20*) In Airin, there are many small bars that can accommodate around 10 customers, although there is no official report. Some TB patients visited bars frequently, but the majority of patients drank alcohol alone in their room. Most importantly, alcohol use impairs the immune system, resulting in increased susceptibility to TB infection and reactivation of latent TB. (*21*-*24*) In addition, one meta-analysis showed that people who drink more than 40 g of alcohol per day and/or have an alcohol use disorder have a substantially elevated risk of active TB. (*24*) In our study, the volume of alcohol intake was not specified. However, for health education, it is important to communicate that reducing alcohol intake could reduce the risk of development of TB.

The main limitation of this study is that the controls were not randomly selected, but they were recruited among supportive house residents who had voluntarily sought health consultations. Therefore, the controls might not be representative of all residents in Airin. The eight supportive houses are located in the subdivision where the TB notification rate has always been the highest in Airin. Thus, the TB notification rate in supportive houses was, in fact, higher than that of the entire Airin area.

An earlier study by the Osaka Social Medical Center (*25*) investigated the nutrient intake of persons on public assistance who attended hospital outpatient departments from July to October 2015. According to the study, 37.6% (47/125) of the respondents drank alcohol, which is not statistically significant (*P* = 0.23) from the controls of our study (31.1%, 59/190). In the same manner, the average BMI of the respondents was 24.9, which is also not statistically significant from the BMI of our controls (24.4). Therefore, the controls in our study may be representative of the residents in Airin.

We conclude that undernutrition and alcohol use are individual characteristics associated with PTB of the residents in Airin, Osaka City. To strengthen the TB control programme further, it is possible to develop new programmes for primary prevention. We already obtained data on BMI, alcohol use and the number of meals a day of residents who participate in the health education programmes in the eight supportive houses. Therefore, as the first step, we would advise residents to reduce their daily alcohol intake and eat an appropriate number of meals, for example, three meals a day, to promote general health and to reduce the risk of development of TB. We also encourage residents with these risks to schedule regular chest X-rays for early case finding of TB disease. After the above model is proven to be effective, the same activity would be applied to other residents in Airin.
